# Predictors of Patient Engagement in Telehealth-Delivered Tobacco Cessation Treatment during the COVID-19 Pandemic

**DOI:** 10.3390/ijerph21020131

**Published:** 2024-01-25

**Authors:** Annemarie D. Jagielo, Amy Chieng, Cindy Tran, Amy Pirkl, Ann Cao-Nasalga, Ashley Bragg, Rachelle Mirkin, Judith J. Prochaska

**Affiliations:** 1 PGSP-Stanford PsyD Consortium, Palo Alto University, Palo Alto, CA 94304, USA; ajagielo@stanford.edu; 2 Department of Psychiatry & Behavioral Sciences, School of Medicine, Stanford University, Stanford, CA 94305, USA; 3 Stanford Prevention Research Center, Department of Medicine, Stanford University, Palo Alto, CA 94304, USA; achieng@stanford.edu; 4 Health Education, Engagement and Promotion, Stanford Healthcare, Menlo Park, CA 94025, USA; cindytran@stanfordhealthcare.org (C.T.); pirkl.amy@mayo.edu (A.P.); acaonasalga@stanfordhealthcare.org (A.C.-N.); abragg@stanfordhealthcare.org (A.B.); rmirkin@stanfordhealthcare.org (R.M.)

**Keywords:** smoking cessation, tobacco treatment, cancer care, quality improvement, COVID-19, pandemic, oncology

## Abstract

Smoking causes one in three cancer deaths and may worsen COVID-19 outcomes. Telehealth tobacco cessation treatment is offered as a covered benefit for patients at the Stanford Cancer Center. We examined predictors of engagement during the COVID-19 pandemic. Data were abstracted from the Electronic Health Record between 3/17/20 (start of pandemic shelter-in-place) and 9/20/22, including patient tobacco use, demographics, and engagement in cessation treatment. Importance of quitting tobacco was obtained for a subset (53%). During the first 2.5 years of the pandemic, 2595 patients were identified as recently using tobacco, and 1571 patients were contacted (61%). Of the 1313 patients still using tobacco (40% women, mean age 59, 66% White, 13% Hispanic), 448 (34%) enrolled in treatment. Patient engagement was greater in pandemic year 1 (42%) than in year 2 (28%) and year 3 (19%). Women (41%) engaged more than men (30%). Patients aged 36–45 (39%), 46–55 (43%), 56–65 (37%), and 66–75 (33%) engaged more than patients aged 18–35 (18%) and >75 (21%). Hispanic/Latinx patients (42%) engaged more than non-Hispanic/Latinx patients (33%). Engagement was not statistically significantly related to patient race. Perceived importance of quitting tobacco was significantly lower in pandemic year 1 than year 2 or 3. Nearly one in three cancer patients engaged in telehealth cessation treatment during the COVID-19 pandemic. Engagement was greater earlier in the pandemic, among women, Hispanic/Latinx individuals, and patients aged 36 to 75. Sheltering-in-place, rather than greater perceived risk, may have facilitated patient engagement in tobacco cessation treatment.

## 1. Introduction

Smoking is the leading preventable cause of death in the United States and globally and causes one in three cancer deaths [1]. Continued smoking after a cancer diagnosis is associated with poor clinical outcomes, such as greater risk for postoperative treatment complications, treatment side effects, the development of a second primary cancer, and mortality [2,3,4,5]. Yet, nearly half of cancer patients continue to smoke following a cancer diagnosis [5]. Additionally, the Coronavirus Disease 2019 (COVID-19) pandemic, a public health crisis marked by over six million deaths internationally [6], has had broad effects on people’s behaviors, including smoking. Tobacco use increases the risk of pulmonary infections due to upper airway damage and impaired pulmonary immune function [7]. Studies of the association between smoking and COVID-19 incidence and disease outcomes have yielded mixed findings [7,8,9].

The COVID-19 pandemic resulted in substantial disruptions to daily functioning as well as monumental cultural and societal changes (e.g., cancellation of travel, business closures, restricted social gatherings). Tobacco use behaviors varied in response to the COVID-19 pandemic. Many people experienced the heightened levels of stress associated with the pandemic as smoking triggers, while others perceived the potential health risks of smoking and COVID-19 as catalysts to quit. Indeed, studies found increased smoking associated with greater stress, isolation, and boredom, while decreased smoking was related to greater perceived health risks of COVID-19, quarantining with non-smokers, and perceiving additional time to commit to quitting [10,11,12,13,14]. Less studied are patterns and predictors of tobacco cessation treatment engagement during the COVID-19 pandemic. Potential added challenges to accessing treatment for tobacco use during the COVID-19 pandemic included stay-at-home mandates, loss of health insurance due to unemployment, and delayed medical care due to disrupted healthcare systems.

Despite the availability of evidence-based tobacco cessation treatments, which more than double the likelihood of quitting smoking successfully [1,15], most attempts to quit using tobacco are unassisted, and success rates remain low. Within community samples, decreased engagement in tobacco treatment has been associated with race [16,17], younger age, and residing at further distances from treatment clinics [16,18]. Older patients generally have more difficulty quitting compared with younger patients [19], yet cigarette smoking is highest among people aged 25–64 years [20]. Findings regarding sex differences in engagement trends are mixed [21]. Within the oncology setting, patients with tobacco-related cancers (e.g., laryngeal, head and neck) have been found to be less likely to engage in tobacco cessation treatment [22,23]. In addition, research has shown patients’ motivation to quit predicts engagement in smoking cessation services as well as smoking cessation success [24].

Understanding the effectiveness of different treatment models, such as “opt-in” and “opt-out,” is critical to improving patient engagement. Tobacco treatment is typically delivered through an “opt-in” approach in which patients are asked if they would like to receive a referral, and treatment is provided only to patients who express a desire to quit smoking [25,26]. The opt-out model of tobacco service delivery is an alternative approach in which all patients are screened for tobacco use and offered care with the option to opt out of treatment. Given that a minority of patients express an immediate readiness to quit smoking, opt-in approaches result in lower evidence-based treatment engagement compared with “opt-out” models of care [26,27]. Opt-in approaches may also pose the risk for clinician referral bias and thus may result in racial and ethnic disparities in tobacco cessation service delivery. For example, prior research [28,29] has found that non-Hispanic White patients are referred to tobacco treatment services, including tobacco cessation counseling and pharmacotherapy, at higher rates than non-Hispanic Black and Hispanic patients. The opt-out model has demonstrated feasibility, acceptability, efficacy, and sustainability for treating tobacco use in the oncology setting [25,30,31].

As part of a National Cancer Institute Moonshot P30 Supplement, the Stanford Cancer Center (SCC) has integrated evidence-based cessation treatment into cancer care, with demonstrated feasibility and efficacy [25,32]. The Cancer Moonshot, established by Congress and funded through the 21st Century Cures Act in 2016, aims to further cancer treatment through research with the long-term goals of reducing the cancer rate death by 50% within the next 25 years [33]. The Cancer Center Cessation Initiative (C3I) was created to expand the reach of evidence-based smoking cessation treatment across NCI-Designated Cancer Centers [34]. A core component of C3I-funded cancer centers includes utilizing a systems-based approach by leveraging the Electronic Health Record (EHR) to integrate tobacco cessation treatment into cancer care [34]. In addition, C3I centers are required to address sustainability by developing strategies to maintain the smoking cessation treatment following the end of funding [34]. Research has demonstrated that C3I-funded cancer centers produce noteworthy quit rates for modest costs [31]. C3I-funded cancer centers have further demonstrated evidence of reducing tobacco-related health disparities by expanding care to all patients with cancer [21].

As with the treatment of other forms of addiction, patient engagement in tobacco treatment services can be challenging. To reduce barriers to access and maximize patient choice, Stanford’s Tobacco Treatment Service offers a menu of telehealth services as a covered benefit for SCC patients and their family members who use tobacco. Treatment options include: (a) cessation medication management; (b) individual, couples, and group counseling; and (c) quitline referral. The interdisciplinary Stanford Tobacco Treatment Service team includes a licensed clinical psychologist, two psychiatrists, a program manager, a patient outcomes evaluator, and four predoctoral practicum students who provide tobacco cessation counseling under the supervision of a licensed clinical psychologist. The telehealth-delivered treatment model, established prior to the COVID-19 pandemic [25], allowed for uninterrupted patient care throughout the pandemic.

Altogether, the SCC treats approximately 33,000 patients annually. With a universal tobacco screening process in place, all patients seen for care at the SCC who report recent tobacco use are contacted by the Stanford Tobacco Treatment Service (opt-out model). The Stanford Tobacco Treatment Service team treats about 400 patients annually. To date (10 August 2023), since January 2019, the Stanford Tobacco Treatment Service has engaged 1637 SCC patients in treatment (44% engagement rate); tobacco quit rates are 27% at 6 months and 34% at 24 months among those with reported outcomes (if those with missing data are assumed to have returned to using tobacco, quit rates are 19% at 6 months and 25% at 24 months). The Stanford Tobacco Treatment Service’s engagement and efficacy outcomes are “best in class” per the medical literature [25,32]. 

This study aims to understand the dynamics of patient engagement in tobacco cessation treatment during a global health crisis, addressing a gap in the literature. Understanding characteristics associated with patient engagement in tobacco cessation treatment further informs quality improvement efforts for patient outreach and service delivery and supports C3I sustainability efforts. As such, the current study sought to understand patient engagement in tobacco cessation treatment during the COVID-19 pandemic. We examined patient engagement during the first 2.5 years of the COVID-19 pandemic in relation to patient age, sex, race, ethnicity, and year of the pandemic and examined treatment preferences among patients who engaged in treatment services. Project activities were reviewed by Stanford’s Research Compliance Office and determined to fall within the domain of a quality improvement evaluation (Protocol #48420).

## 2. Materials and Methods

To examine the impact of the COVID-19 pandemic on patient treatment engagement, data were abstracted from the EHR from 3/17/20 (the start of the shelter-in-place order in the Bay Area) to 9/20/22. Patient demographic data included sex, age, race, and ethnicity. Patient disposition was coded as unreached, already quit, declined, or engaged in treatment. Engagement was broadly defined to include engagement in any of the following types of evidence-based cessation treatment: (1) individual, group, or couple’s counseling, (2) medication management, and (3) quitline referral. The disposition date was coded as pandemic year 1, 2, or 3. Race was coded into four categories: White, Black, Asian/Pacific Islander, and other. Ethnicity was coded as non-Hispanic/Latinx, Hispanic/Latinx, and declined to state/other. Patient age was categorized as: 18–35, 36–45, 46–55, 56–65, 66–75, and 76 and older. 

The importance of quitting was obtained from a subset of patients, at the time of first contact with the tobacco treatment specialist. The importance of quitting was assessed with a single item, “How important is it for you to quit now?” and ranged from 1 (lowest importance of quitting) to 10 (highest possible importance of quitting). These data were limited to a subset of patients due to patient factors (e.g., availability for an extended initial outreach phone call) and variability in the team members making the initial outreach attempt.

Descriptive and logistic regression analyses were performed in SPSS 27.0. The data followed a normal distribution and did not contain outliers. The alpha level was set at 0.05. A multivariate logistic regression was run to examine whether sex, age, race, ethnicity, and pandemic year predicted patient treatment engagement. Chi-square analyses were conducted to examine treatment service preferences among patients who engaged testing for differences by patient demographics and pandemic year. Finally, we ran a Welch’s one-way ANOVA test, which does not assume equal variances, to examine differences in patients’ ratings of the importance of quitting tobacco by pandemic year. 

## 3. Results

### 3.1. Patient Characteristics

**Figure 1:**
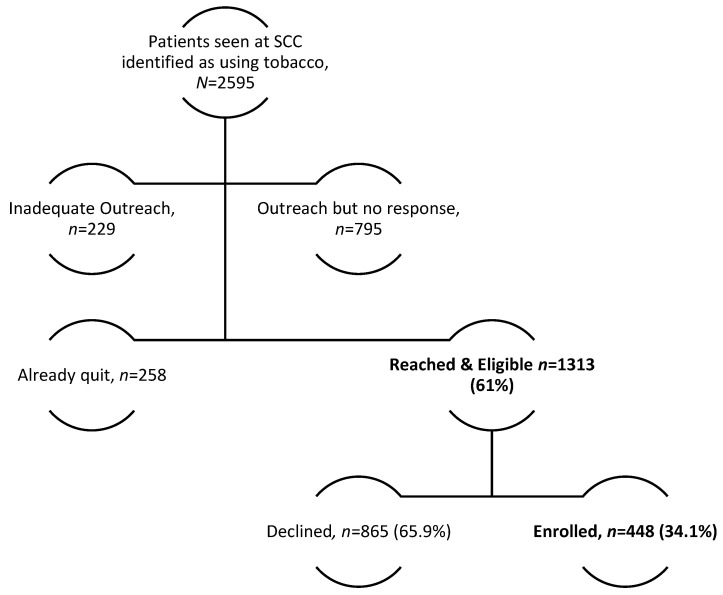
Flow Diagram of Stanford Cancer Center (SCC) Patient Recruitment and Engagement.

During the first 2.5 years of the pandemic, 2595 patients were seen in the SCC as part of routine cancer care and identified in the EHR as recently using tobacco. Among these patients, 795 did not respond to the initial outreach attempt, and 229 were not contacted by a Stanford team member three times per our outreach recommendations. In total, 1571 patients were reached by a tobacco treatment specialist (61%). Of the patients contacted, 1313 reported still using tobacco (40% women, mean age 59, 63% White, 13% Hispanic), and 448 (34%) enrolled in treatment (Figure 1, Table 1). Among the 1008 patients with insurance status recorded, 43.5% had Medi-Cal, 19% Medicare, 34.8% a private plan, and 2.7% other. Among the 1023 patients contacted and eligible with tobacco type recorded, 83.4% reported smoking cigarettes; 5.6% smoked cigars; 7.5% used smokeless tobacco; and 3.5% reported other tobacco use, including e-cigarette (1.5%), pipe (0.5%), hookah (0.2%), and dual-use (1.2%). 

### 3.2. Predictors of Patient Engagement

In the multivariate logistic regression predicting patient engagement, pandemic year and patient sex, age, and ethnicity were statistically significant predictors; race was not (see Table 2). Engagement was significantly greater in pandemic year 1 (42%) compared to year 2 (28%) and year 3 (19%). Engagement was greater for women (41%) than men (30%). Engagement was greater for patients aged 36–45 (39%), 46–55 (43%), 56–65 (37%), and 66–75 (33%) compared to patients aged 18–35 (18%) and >75 (21%). Hispanic/Latinx patients were more likely to engage (42%) compared to non-Hispanic/Latinx patients (33%).

### 3.3. Predictors of Treatment Preferences among Patients Who Engaged

Among patients who engaged in treatment, 59% opted for medication only, 29% opted for counseling only, and 12% opted for both. Separate chi-square tests were run to examine engagement in counseling and engagement in medication consultation by patient gender, age group, race, ethnicity, and pandemic year. Women (47%) were more likely than men (35%) to engage in counseling, χ^2^_(2, *N* = 453)_ = 7.33, *p* = 0.007, while men (77%) were more likely than women (66%) to choose medication management, χ^2^_(2, *N* = 453)_ = 6.62, *p* = 0.010. In pandemic year 1, patients were more likely to pursue pharmacotherapy (86%) compared with pandemic year 2 (44%) and pandemic year 3 (52%), χ^2^_(2, *N* = 453)_ = 83.34, *p* < 0.001, and were less likely to engage in behavioral counseling (26%) compared to pandemic year 2 (70%) and year 3 (59%), χ^2^_(2, *N* = 453)_ = 75.53, *p* < 0.001. Non-Hispanic/Latinx patients were more likely to pursue behavioral counseling (44%) than Hispanic/Latinx patients (26.2%), χ^2^_(1, *N*=436)_ = 7.78, *p* = 0.006. Engagement with medication management did not differ by patient ethnicity, race, or age, and counseling engagement did not differ by race or age.

### 3.4. Perceived Importance of Quitting Tobacco

**Figure 2:**
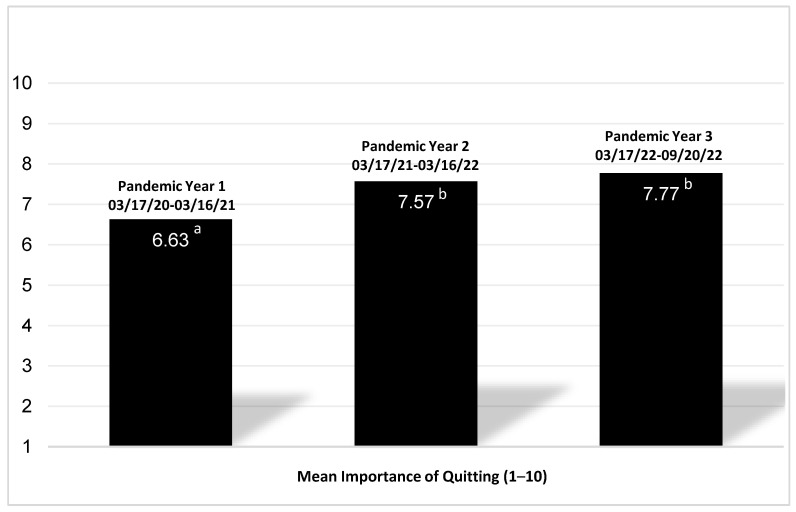
Mean Patient Reported Importance of Quitting by Pandemic Year (*n* = 693). ^1^ The letters indicate which means are different from each other. ^2^ The importance of quitting scale ranges from 1 (lowest) to 10 (highest).

Patient-reported importance of quitting tobacco was obtained in a subset of patients (*n* = 693). A Welch’s ANOVA was conducted, as the homogeneity of variance assumption was not met. Patient ratings of importance differed significantly by pandemic year, F_(2,693)_ = 8.511, *p* < 0.001. In Games–Howell’s post hoc with equal variances not assumed, the difference was significant for pandemic year 1 (M = 6.63, SD = 3.260) compared to pandemic year 2 (M = 7.57, SD = 2.845) and pandemic year 3 (M = 7.77, SD = 2.331), but not for pandemic year 2 and year 3 compared to each other (Figure 2).

## 4. Discussion

Among patients seen for cancer care and contacted by the Stanford Tobacco Treatment Service, engagement across the first 2.5 years of the COVID-19 pandemic was 34%, which is comparable to the service’s 33% engagement rate reported for 2019–2020 [25]. This engagement rate is noteworthy given the operational challenges posed by the pandemic, suggesting the resilience and adaptability of our telehealth services. Similarly, colleagues at Case Western’s Comprehensive Cancer Center recently reported a 33% engagement rate with at least one counseling session as part of their Tobacco Intervention and Psychosocial Support initiative [16]. In contrast, an analysis of 13 NCI-designated cancer centers [35] reported 25% engagement in tobacco cessation treatment, and earlier studies reported 20% engagement rates for comprehensive tobacco treatment services within oncology settings [36,37]. As the COVID-19 pandemic restricted in-person healthcare and strained healthcare systems, our results demonstrate the feasibility of telehealth-delivered tobacco treatment within the oncology setting in mitigating these barriers. These results underscore the potential of telehealth to not only maintain but potentially enhance patient engagement in times of healthcare system strain. The findings also contribute to evidence of C3I sustainability efforts [30].

Finer analysis by pandemic year indicated that engagement was greater in the first year of the pandemic (42%) compared to later. Sheltering-in-place may explain this observation, as individuals may have had more free time to commit to health-related goals and may have been more accessible by phone. The COVID-19 pandemic led to a home fitness revolution, with many individuals committing to a fitness regimen [38]. Perhaps individuals felt similarly motivated to undertake a tobacco cessation commitment. In addition, many people reported an increase in smoking during the COVID-19 pandemic [10,11,14]. It is possible that the engagement trends observed reflect patients’ desire to quit or to reduce their smoking following an increase in smoking early on in the pandemic. Our finding that patient self-reported importance of quitting was the lowest during pandemic year 1 compared to pandemic years 2 and 3, respectively, suggests that greater perceived risk may not have facilitated this engagement trend. Rather, it seems that more patients lower on the importance of quitting spectrum were receptive to engaging in treatment services during the first year of pandemic restrictions. The association between greater COVID-19 risk perceptions and self-reported importance of quitting has been made in prior research [10]. Yingst et al. [10] hypothesized that greater risk perceptions of COVID-19 would lead to increased engagement in cessation behaviors. Our findings suggest that other factors (e.g., sheltering in place) may have been more salient than respiratory risk perceptions of COVID-19 in driving tobacco cessation engagement rates.

The higher observed engagement in pandemic year 1 may also be explained by a sampling issue. Patients reporting tobacco use at an SCC visit are identified in the EHR for outreach by the Stanford Tobacco Treatment Service. Patients receiving cancer care early on in the pandemic, when many medical care services were delayed [39], likely had high acuity medical needs and may, due to their cancer disease state, have been more receptive to tobacco cessation treatment. For example, many SCC patients are advised to quit smoking prior to cancer-related surgical procedures (i.e., considered essential medical procedures during the COVID-19 pandemic). It is possible that these patients had a greater medical urgency to quit, leading to increased engagement in treatment. Future studies should examine the impact of disease severity on patient engagement in tobacco treatment services. 

While patients eligible for tobacco treatment services were more likely to be men, we observed higher rates of tobacco treatment engagement among women. While past research examining sex differences in smoking cessation treatment has yielded mixed findings, our results are consistent with research suggesting that women engage in cessation treatment at higher rates than men [21]. Further, we found that among patients who engaged, women were more likely to engage in cessation counseling, while men were more likely to elect medication management. In the US, men are more likely to use tobacco than women [40]. For mental health treatment, women are more likely to engage in counseling than men [41]. Going forward, qualitative research should explore factors that may contribute to these engagement trends and examine potential barriers to engaging men in tobacco cessation treatment. Though patients in our service rarely, if ever, express a gender identity preference with regard to treatment provider, our team includes counselors and prescribers of different gender identities and would be able to accommodate such requests. 

We also found that engagement was higher among patients aged 36 to 75 compared with younger (18–24) and older (76+) patients. Young adults (ages 18–24) tend to underuse evidence-based tobacco cessation treatments and often make unassisted quit attempts [21,42]. This trend might reflect generational differences in attitudes towards tobacco cessation and telehealth, with younger adults possibly facing unique challenges such as the rapid transition to remote learning and work. Our findings highlight the crucial need to enhance the outreach and support offered to young adults who use tobacco to increase their engagement in tobacco cessation services. 

Regarding older adults, a large population-based study [43] found that individuals older than 75 were less interested in quitting smoking than adults of other ages. Older people who smoke may face unique barriers to quitting tobacco. For example, some older adults believe that there is no point in quitting after a lifetime of smoking, as the “damage is already done” [44,45,46]. In addition, many older adults cited barriers such as low self-efficacy and doubt regarding the negative health effects of smoking [46]. Older adults who use tobacco have also expressed skepticism regarding cessation medications, despite their evidence [46]. It is possible these beliefs were exacerbated by information regarding the risks associated with contracting COVID-19. Telehealth-delivered services may pose a barrier for older patients with limited technological proficiency [47]. Older adults represent a high-risk group for medical complications related to smoking and contraction of COVID-19 infection. Given this, more research is needed to identify strategies to increase tobacco cessation treatment engagement in this age cohort. Moreover, patients in the 35–75 age range may have unique reasons to engage in tobacco cessation treatment that should be explored in future qualitative studies. 

Our results demonstrate that Hispanic/Latinx patients were more likely to engage in our tobacco cessation services compared to non-Hispanic/Latinx patients. This is notable, given that minority populations are traditionally less likely to be referred for and utilize evidence-based cessation treatment compared with non-Hispanic White patients [28,29,48]. This trend suggests that minority groups may be eager to engage in evidence-based cessation services when services are accessible. We did not observe significant differences in patient engagement by patient race. Our program promotional materials are available in eight different languages, and our team is able to provide care in over 200 spoken languages with the support of interpreter services, which are available 24/7. A systems-level opt-out strategy, especially one that offers tobacco cessation treatment as a covered benefit and via telehealth, appears successful for engaging diverse patients and potentially reducing tobacco-related health disparities and enhancing equity. 

We found that patients were more likely to pursue pharmacotherapy than behavioral counseling. Utilizing pharmacotherapy and counseling combined is the most effective strategy to successfully achieve tobacco cessation [49]. We now emphasize the value of combination treatment to a greater extent in program materials and outreach calls and revisit opportunities to engage in medication management and counseling with patients over time. Coordinated care and communication among service prescribers and counselors is also facilitated. 

Lastly, we observed differences in patient treatment preferences throughout the COVID-19 pandemic. In pandemic year 1, patients were significantly more likely to pursue pharmacotherapy and less likely to engage in counseling compared to later pandemic years. It is possible that stay-at-home mandates restricted retail access to tobacco products, resulting in patients requesting cessation medication, such as nicotine replacement therapy, at higher rates. The increase in patients selecting behavioral counseling, from 26% in pandemic year 1 to 70% in pandemic year 2, may partially be explained by a staffing change in the team member making the initial outreach calls to present the program offerings. Hispanic/Latinx patients who engaged in services were significantly less likely to select behavioral counseling compared to non-Hispanic/Latinx patients. Prior research has found that Hispanic/Latinx individuals tend to seek mental health treatment less frequently than non-Hispanic/Latinx patients [50]. In our outreach, in addition to offering translation services so that counseling is available in the patients’ language of preference, we underscore the centrality of cultural competence in our tobacco cessation counseling sessions. Taken together, these findings underscore the importance of quality improvement and quality assurance work in monitoring factors that influence patient engagement in treatment services. 

As a quality improvement analysis, study findings may not generalize to other settings. Of interest would be comparison to other cancer centers throughout the United States and consideration of additional sociodemographic factors and mental health comorbidities. The collection of patient sex data was limited to information in the EHR and binary. Future research should collect self-reported gender identity to include gender-expansive populations. With our data collection, pandemic year 3 was a half-year; however, with 152 patients identified as eligible, numbers were sufficient for inclusion. The importance of quitting was collected in a subset of patients, which reduced power and may introduce bias in the analysis.

## 5. Conclusions and Future Directions

In summary, these findings suggest that telehealth-delivered tobacco services were of interest to patients during the COVID-19 pandemic. Patients were more likely to engage earlier in the pandemic compared to later pandemic years. Engagement was more likely among women than men, Hispanic/Latinx patients compared to non-Hispanic/Latinx patients, as well as patients aged 36 to 75 compared to patients 18–35 and 75 years and older. Findings elucidate the importance of leveraging quality improvement efforts to enhance patient outreach and ultimately increase patient engagement in treatment services. To identify barriers and facilitators to patient engagement, qualitative interviews are underway with patients who did and did not engage in tobacco cessation treatment. Ultimately, we aim to implement findings into our outreach efforts to enhance patient engagement, support tobacco cessation, and improve cancer care outcomes. 

## Figures and Tables

**Table 1:** Demographic Characteristics of Patients Reached and Identified as Eligible (*n* = 1313) and among those who Enrolled (*n* = 448) in Tobacco Treatment Services.

	All Eligible Patients (*n* = 1313)		Enrolled Patients (*n* = 448)	
Patient Demographics	*n* (%)	Mean (SD)	*n* (%)	Mean (SD)
Age (years)		59.4 (14.4)		58.8 (12.5)
Sex				
Male	787 (59.9%)		232 (51.8%)	
Female	526 (40.1%)		216 (48.2%)	
Race				
White	812 (62.8%)		283 (63.2%)	
Black	97 (7.5%)		29 (6.5%)	
Asian/Pacific Islander	141 (10.9%)		41 (9.2%)	
Missing/Other	263 (18.7%)		95 (21.2%)	
Ethnicity				
Non-Hispanic/Latinx	1085 (82.6%)		360 (80.4%)	
Hispanic/Latinx	170 (12.9%)		71 (15.8%)	
Missing	58 (4.4%)		17 (3.8%)	

**Table 2:** Multivariate Logistic Regression: Predictors of Patient Engagement in Tobacco Treatment (*n* = 1313).

Variable	% Engaged	β	SE	Wald’s χ^2^	df	*p*	Exp (B)
Age				28.27	5	<0.001	
Ages 18–35 (Ref)	18%						
Ages 36–45	39%	1.072	0.332	10.43	1	0.001	2.92
Ages 46–55	43%	1.214	0.318	14.60	1	<0.001	3.37
Ages 56–65	37%	0.966	0.306	9.98	1	0.002	2.63
Ages 66–75	33%	0.801	0.307	6.82	1	0.009	2.23
Ages 76+	21%	0.184	0.352	0.27	1	0.601	1.20
Sex							
Male (Ref)	30%						
Female	41%	0.471	0.124	14.40	1	<0.001	1.60
Race				2.33	3	0.507	
White (Ref)	35%						
Black	30%	−0.361	0.244	2.20	1	0.138	0.70
Asian American	29%	−0.111	0.211	0.28	1	0.599	0.90
Missing/Other	36%	−0.044	0.185	0.06	1	0.813	0.96
Ethnicity				5.37	2	0.068	
Non-Hispanic (Ref).	33%						
Hispanic	42%	0.405	0.205	3.89	1	0.049	1.50
Missing	29%	−0.233	0.325	0.51	1	0.474	0.79
Pandemic Year				38.99	2	<0.001	
Year 1 (Ref)	42%						
Year 2	28%	−0.648	0.133	23.75	1	<0.001	0.52
Year 3	19%	−1.1092	0.226	23.42	1	<0.001	0.34
Constant		−1.367	0.302	20.53	1	<0.001	0.26

## Data Availability

The fully de-identified data described in the article may be available upon reasonable request from the corresponding author [J.J.P.].
